# Density Dependence Influences the Efficacy of Wastewater Remediation by *Lemna minor*

**DOI:** 10.3390/plants10071366

**Published:** 2021-07-03

**Authors:** Éamonn Walsh, Neil E. Coughlan, Seán O’Brien, Marcel A. K. Jansen, Holger Kuehnhold

**Affiliations:** 1 School of Biological, Earth and Environmental Science, University College Cork, Distillery Fields, North Mall, T23N73K Cork, Ireland; neil.coughlan@ucc.ie (N.E.C.); 115449682@umail.ucc.ie (S.O.); m.jansen@ucc.ie (M.A.K.J.); 2 Environmental Research Institute, University College Cork, Lee Road, T23XE10 Cork, Ireland; 3 Department of Ecology, Leibniz Centre for Tropical Marine Research (ZMT), 28359 Bremen, Germany; holger.kuehnhold@leibniz-zmt.de

**Keywords:** duckweed, wastewater, remediation, density, surface cover, circular economy, lemna

## Abstract

As part of a circular economy (CE) approach to food production systems, *Lemnaceae*, i.e., duckweed species, can be used to remediate wastewater due to rapid nutrient assimilation and tolerance of non-optimal growing conditions. Further, given rapid growth rates and high protein content, duckweed species are a valuable biomass. An important consideration for duckweed-mediated remediation is the density at which the plants grow on the surface of the wastewater, i.e., how much of the surface of the medium they cover. Higher duckweed density is known to have a negative effect on duckweed growth, which has implications for the development of duckweed-based remediation systems. In the present study, the effects of density (10–80% plant surface coverage) on *Lemna minor* growth, chlorophyll fluorescence and nutrient remediation of synthetic dairy processing wastewater were assessed in stationary (100 mL) and re-circulating non-axenic (11.7 L) remediation systems. Overall, *L. minor* growth, and TN and TP removal rates decreased as density increased. However, in the stationary system, absolute TN and TP removal were greater at higher densities (50–80% coverage). The exact cause of density related growth reduction in duckweed is unclear, especially at densities well below 100% surface coverage. A further experiment comparing duckweed grown at ‘low’ and ‘high’ density conditions with the same biomass and media volume conditions, showed that photosynthetic yield, Y(II), is reduced at high density despite the same nutrient availability at both densities, and arguably similar shading. The results demonstrate a negative effect of high density on duckweed growth and nutrient uptake, and point towards signals from neighbouring duckweed colonies as the possible cause.

## 1. Introduction

Globally, the provision of nutritious food is a challenging endeavour [1,2]. Climate change, a reduction in the *per capita* availability of arable land, as well as soil erosion, chemical overuse and finite resources have decreased food security [3,4,5,6]. In recent years, the adoption of circular economy (CE) principles in food production systems has been suggested as a mechanism to improve resource-efficiency and the sustainability of food production [7]. In essence, CE promotes long-term retention and reuse of resources, as well as minimisation of waste generation, resulting in a reduced need for raw materials [8]. Thus, CE principles encourage the adoption of closed-loop production patterns, whereby waste is appropriated as a resource [8], reducing emissions and energy consumption in the process [9].

Dairy products are a major and important source of nutrition, employment and trade worldwide [10,11]. However, large volumes of wastewater are created as a consequence of dairy production and processing. It is estimated that up to 10 L of wastewater is created per litre of milk processed, making dairy processing waste one of the most significant waste streams in the food industry [12]. Dairy processing wastewaters tend to contain particularly high concentrations of organic matter, measured as chemical oxygen demand (COD): 2000–6000 mg L^−1^ COD [13]; 4420 mg L^−1^ COD [14]; 55,430–70,150 mg L^−1^ COD [15]. Moreover, these wastewaters generally contain high concentrations of nutrients, especially ammonium (64–270 mg L^−1^ NH_4_-N), nitrate (9–30 mg L^−1^ NO_3_-N) and phosphate (20–356 mg L^−1^ PO_4_-P) [13,15,16]. The disposal of such wastewater often lacks value-capture in the treatment process [17]. For example, valuable nitrogen-containing nutrients such as nitrate and ammonium are commonly released as gaseous N_2_ [12]. Phosphate is typically precipitated using aluminium chloride, lime and similar additives, to generate a precipitate sludge [18]. The resulting non-soluble form of phosphate has arguably limited further benefit as a fertiliser [19]. 

Phytoremediation has been proposed as a viable alternative to traditional wastewater treatments, as phytoremediation removes plant nutrients from wastewaters and also retains these elements in a chemical form suitable for further use [20,21]. Dairy processing wastewater is considered to be a good candidate for phytoremediation as it generally contains an abundance of essential plant nutrients, such as ammonium, nitrate and phosphate [22]. 

Duckweed, *Lemnaceae*, are a family of floating aquatic plants with excellent potential for phytoremediation due to a tolerance of wastewater conditions [23,24,25], fast growth rates [26] and high protein or starch content [27,28], as well as demonstrated use as feed, food and biofuel [29,30,31]. Thus, these plants can combine efficient wastewater remediation with the creation of a valuable plant biomass. To date, few studies have attempted to assess the suitability of duckweed for remediation of dairy processing wastewater. However, in principle duckweed has been shown to remediate dairy processing wastewater that lacks organic components, such as sugars and fats [32]. As a high proportion of these organic components are generally removed by existing microbial-based treatment technologies, such as sequential batch reactors or anaerobic digesters [33,34], the incorporation of duckweed into the remediation process is a realistic approach.

Wastewater remediation by duckweed is a surface process, whereby a layer of duckweed takes up nutrients from the underlying water column. In their natural habitats, most duckweed species grow in dense, floating mats [35]. Once mats have filled the available space, individual colonies begin to overlap and shade each other. Such highly crowded conditions negatively impact duckweed growth rates [36,37], and duckweed may even start to senesce and release nutrients back into the water column [38]. Conversely, a higher duckweed plant density can increase the potential for uptake of nitrogen and phosphorus [39]. Given the implications for biomass production and wastewater remediation [40], an improved understanding of the relationship between plant surface density and biomass yield, as well as net nutrient uptake is required. Earlier work has shown that a low growth rate at a high plant density does not necessarily imply a low biomass yield or low N and P removal [37]. Accordingly, to achieve effective phytoremediation, determination of optimal duckweed density for nutrient removal, plant growth and biomass yield per water surface area is required. In the present study, the effects of density on duckweed growth and remediation were quantified. This was done under axenic conditions, using stationary tanks containing either synthetic dairy processing wastewater or an optimal medium (half-strength Hutner’s). Furthermore, with the aim of reproducing some of the conditions of large-scale duckweed phytoremediation systems, *L. minor* was cultivated on synthetic dairy wastewater using a larger scale non-axenic re-circulatory system. The results will inform management of duckweed-based remediation systems.

## 2. Materials and Methods

### 2.1. Stock Cultivation

The duckweed strain used in this study was *Lemna minor* L.–Blarney, strain number 5500 in the Rutgers Duckweed Stock Cooperative database [41]. A sterile stock of *L. minor* was cultivated on half-strength Hutner’s medium [42] under an average light intensity of 50 µmol m^−2^ s^−1^ photosynthetically active radiation (PAR) in a controlled growth-room (22 °C, 14 h:10 h light:dark photoperiod). 

### 2.2. Experimental Design

#### 2.2.1. Synthetic Dairy Processing Wastewater

The synthetic dairy processing wastewater used in this study is based on the composition of real dairy processing wastewater found in dairy wastewater treatment facilities [43], with modifications as detailed in Walsh et al. [21]. The pH was reduced to, and maintained at, around 5.0 from a natural value of 8 with 1 M H_2_SO_4_ to facilitate optimal *L. minor* growth [44]. H_2_SO_4_ was chosen to decrease pH as SO_4_-S has a wide ‘optimal’ range (0.5–20 mM) in which it does not cause adverse or beneficial effects towards duckweed, with a high maximum tolerated concentration of 60 mM, as per Walsh et al. [21].

#### 2.2.2. Manipulation of Plant Density

In this paper, the term “plant density” is used to refer to the relative surface cover of the medium by *Lemna minor*, i.e., the proportional cover by duckweed as a fraction of the total available surface area. Plant density, i.e., surface cover, is linked to plant biomass per m^2^. Plant biomass always refers to fresh duckweed biomass. Plant density was either measured directly, or estimated based on biomass per surface area. Direct density measurements were performed using the imaging software Easy Leaf Area [45] which distinguishes duckweed frond surface cover from non-duckweed covered surface area. This non-invasive technique could be used throughout the duration of an experiment. Alternatively, plant density, i.e., relative surface cover, was estimated based on biomass per m^2^ of surface area. In this scenario, the latter values were calculated using a calibration curve for *L. minor* biomass versus surface area. To generate a calibration curve, a number of colonies were taken at random from a stock culture acclimated to the relevant medium. The total surface area and mass of these ‘representative’ colonies were measured and the area/mass ratio was calculated.

#### 2.2.3. Stationary Remediation Experiment 1: Growth and Remediation at Variable Plant Densities

**Figure 1:**
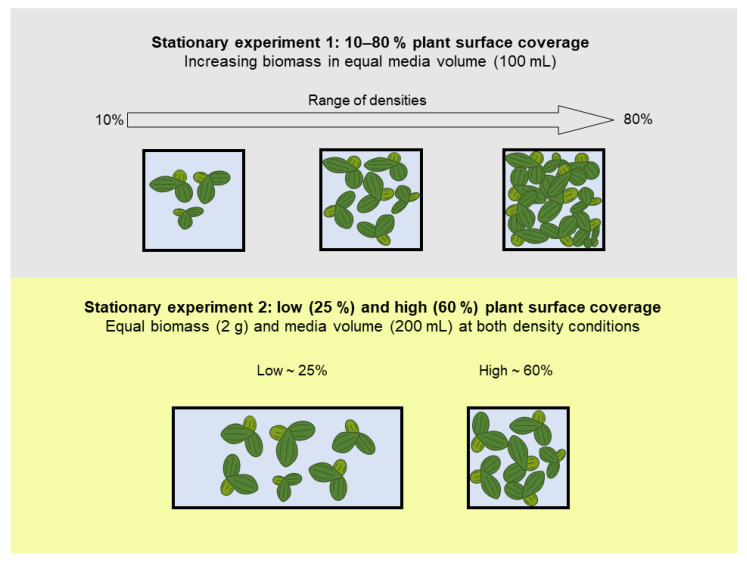
Set-up of stationary remediation experiments 1 and 2.

Two stationary experiments were conducted. In the first, scoping, experiment *L. minor* was grown on 100 mL of synthetic dairy wastewater for seven days (days 0–7) using a range of eight density conditions (10, 20, 30, 40, 50, 60, 70, 80% plant coverage of total surface area, *n* = 6; Figure 1). The corresponding biomass per container surface area (ranging from 21 to 154 g m^−2^) was estimated based on a mass/area ratio of *L. minor* biomass. Plants were kept in Magenta vessels (GA-7, surface area (SA) 42.24 cm^2^) in a controlled growth room (average light intensity 50 µmol m^−2^ s^−1^ PAR, 22 °C, 16 h:8 h light:dark photoperiod). To start experiments, *L. minor* colonies were taken at random from stock cultures that had been acclimated to synthetic wastewater for seven days. The range of density conditions was created by adding varying numbers of *L. minor* colonies and determining the total frond surface cover using Easy Leaf Area imaging. Plant densities were maintained at ±2% of target surface cover throughout the experiment by removing excess plant material every 2–3 days, and this process was guided by measurements of frond surface area, as determined by Easy Leaf Area. Excess plant biomass removed throughout the experiment was weighed and used to calculate specific growth rate (SGR) and relative growth rate (RGR) (*n* = 6, except for 40% where *n* = 4). Total nitrogen (TN) and total phosphorous (TP) were measured from medium samples taken on days 0 and 7 (*n* = 6, except for 40% where *n* = 4). Protein content was measured from plant samples taken on day 7 (*n* = 6, except for 40% where *n* = 4).

#### 2.2.4. Stationary Remediation Experiment 2: Growth and Remediation at Low and High Density

In the second stationary experiment, the negative effect of higher duckweed density on growth was explored in greater detail. To achieve this, two density conditions were created by using two containers with different surface areas, but containing the same medium volume (200 mL) and initial plant biomass (2 g; Figure 1). For both density conditions, *L. minor* was grown for seven days (days 0–7) on either synthetic wastewater or half-strength Hutner’s medium. The experiment was conducted in a controlled growth room (average light intensity 50 µmol m^−2^ s^−1^ PAR, 22 °C, 16 h:8 h light:dark photoperiod). The plant density conditions consisted of low (25%; 193 g m^−2^) and high (60%; 476 g m^−2^) plant coverage of the total surface area (*n* = 4 per experimental treatment). The % density was determined using Easy Leaf Area imaging, while the density in g m^−2^ was calculated based on the ratio of the weighted inoculum (2 g) and the container surface area. The two densities were created by using two types of growing containers (Magenta vessels with 42.25 cm^2^ SA for 60% cover and larger circular glass containers with 103.87 cm^2^ SA for 25% cover). In both cases, 2 g of colonies were selected randomly from stock cultures, which had already been acclimated to their respective media for seven days within the controlled growth room. Densities were maintained at ±2% of target surface cover throughout the experiment through the removal of excess plant material every 2–3 days, a process guided by measurements of frond surface area, as determined by Easy Leaf Area. Excess plant biomass removed throughout the experiment from each replicate was weighed and used to calculate SGR and RGR (*n* = 4). Chlorophyll *a* fluorescence measurements were taken on randomly selected plants on days 0 and 7 (*n* = 4). TN and TP were measured from medium samples taken on days 0 and 7 (*n* = 4). In both stationary experiments, any water loss due to evaporation was countered by adding deionised water to maintain original volumes.

#### 2.2.5. Re-Circulating Remediation System: Growth and Remediation at Variable Plant Densities

To determine the effect of plant density on duckweed growth and remediation capacity under more realistic operating conditions, *L. minor* was grown in a non-axenic, re-circulating system containing 11.7 L of synthetic dairy wastewater, for five days (days 0–5), and at three densities (20, 50 and 80% plant coverage of total surface area of 600 cm^2^, *n* = 4). Plant density was measured using Easy Leaf Area, whilst the corresponding biomass per container surface area (ranging from 50 to 187 g m^−2^) was estimated based on a mass/area ratio of *L. minor* biomass. The experiment was conducted within a controlled environment room (300 µmol m^−2^ s^−1^ PAR, 16 h:8 h light:dark photoperiod). In this experimental system, synthetic wastewater was re-circulated between two tanks, an upper duckweed tank and a lower sump tank at a rate of 125 L per hour. The upper tanks of each replicate treatment were seeded at their respective plant surface densities on the initial day of the experiment, using stock plants acclimated to synthetic wastewater for seven days. Excess biomass grown over the course of the experiment was removed twice over the five-day experiment to maintain densities within ±2% of target surface coverage, as determined using Easy Leaf Area imaging. Excess plant biomass removed throughout the experiment from each replicate was weighed and used to calculate SGR (*n* = 4). TN and TP were measured from medium samples taken on days 0 and 5 (*n* = 4). Protein content was measured from plant samples taken on day 5 (*n* = 4).

### 2.3. Measured Parameters

#### 2.3.1. Growth

All plant biomass was dried with absorbent tissue-paper to remove excess water and ensure reliable measurements before weight measurements. A specific growth rate (SGR), for growth comparisons within the present study, was calculated from estimations and measurements of fresh biomass using the formula [46]:

(1) $$SGR=\frac{W_{2}/W_{1}}{\Delta T}$$

where *W*_1_ is starting mass, *W*_2_ is the increase in mass over the course of the entire experiment and Δ*T* is the length of the experiment. Except for stationary experiment 2, starting mass (*W*_1_) was estimated rather than measured directly and this was guided by a calibration curve of biomass versus plant surface area. 

For comparison with literature sources, a relative growth rate (RGR) was calculated from estimations and measurements of fresh biomass using the formula [47]:

(2) $$RGR=\frac{ln\frac{W_{3}}{W_{1}}}{\Delta T}$$

where *ln* is the natural log, *W*_1_ is starting biomass, *W*_3_ is total biomass on day 3 and Δ*T* is the length of time. As biomass was removed throughout each experiment to maintain a constant plant density, this formula was only used to calculate the *RGR* up to the first instance of removal (day 3). The total increase in mass over the course of the experiment is presented as the yield.

#### 2.3.2. Chlorophyll *a* Fluorescence

Chlorophyll *a* fluorescence measurements were taken for randomly selected plants on days 0 and 7, using a pulse amplitude modulated fluorometer (WALZ Imaging fluorometer, Effeltrich, Germany). The procedure that was followed is detailed in Walsh et al. [32].

#### 2.3.3. Total Nitrogen and Total Phosphorous Analysis

A sample of medium was taken for total nitrogen (TN) and total phosphorous (TP) analysis on the initial and final days of each experiment. For TN analysis, Hach test LCK138 was used with a Hach DR3900 spectrophotometer. Firstly, the sample was digested with peroxo-disulphate for one hour at 100 °C causing inorganically and organically bonded nitrogen to oxidise to nitrate (Koroleff digestion). The resulting oxidised nitrate was then analysed photometrically in a reaction with 2,6-dimethylphenol. For TP analysis, Hach test LCK348 was used. Firstly, the medium was digested using the persulphate digestion method for one hour at 100 °C. The resulting solution was then analysed photometrically through the ascorbic acid/phosphomolybdenum blue method. 

#### 2.3.4. Protein Analysis

*Lemna minor* samples, taken on the final day of experiments, were kept at −20 °C until used for protein extraction and analysis. Protein was extracted using 50 mM potassium phosphate buffer (pH 7, containing 0.1 mM polyvinylpyrrolidone (PVP) and 0.1 mM EDTA). Between 50–80 mg of fresh plant material was homogenised in cold potassium phosphate buffer (1 mL of buffer to 80 mg of plant sample). The homogenised sample was then centrifuged at 20,000× *g* for 30 min at 4 °C [48]. The resulting supernatant was used for protein analysis using the Bradford method with bovine serum albumin as a standard [49]. For absorbance measurements, 5 µL of sample was added to 1 mL of Bradford reagent in a cuvette and left for five minutes in dark conditions. Absorbance was measured at 595 nm using a spectrophotometer (UV-160A Shimadzu). In order to calculate the proportion of protein based on dry plant biomass, 4% dry weight content of fresh duckweed weight was used [28].

### 2.4. Data Analysis

Statistical analyses were conducted using R (version 3.4.3 [50]). One- and two-way ANOVAs were used to analyse differences between treatments for the measured parameters. Post hoc Tukey tests were used for pairwise comparisons of treatment groups. Normality was assessed through a graphical assessment of the distribution of the residual values for data points (i.e., histogram). Homoscedasticity was assessed with ‘residuals vs. predicted values’ plots as well as Fligner-Killeen and Levene’s tests. 

## 3. Results

### 3.1. Stationary Remediation Experiment 1: Growth and Remediation at Variable Plant Densities

**Figure 2:**
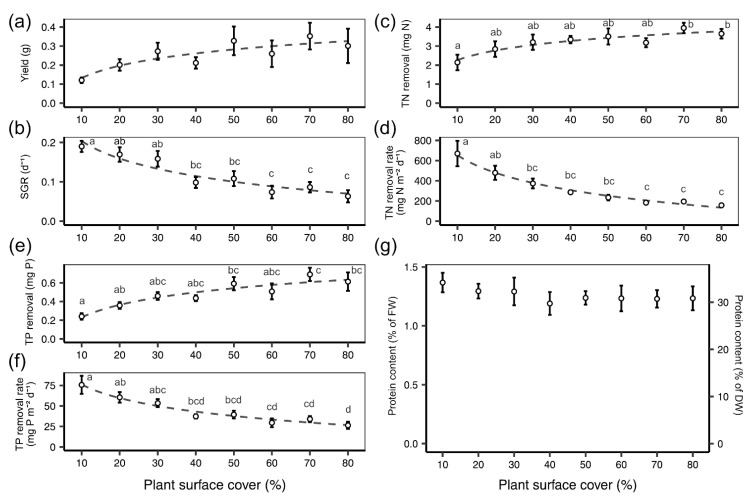
Mean (±SE) values with natural log trendline of (**a**) yield (g), (**b**) specific growth rate (SGR, d^−1^), (**c**) total nitrogen (TN) removal (mg N), (**d**) TN removal rate per frond surface area (mg N m^−2^ d^−1^), (**e**) total phosphorous (TP) removal (mg P), (**f**) TP removal rate per frond surface area (mg P m^−2^ d^−1^) and (**g**) % protein content based on fresh duckweed biomass, FW, and dry duckweed biomass, DW, for *L. minor* grown on synthetic wastewater over 7 days under a range of plant surface covers (10–80%). Points that do not share the same letter significantly differ from one another, as per the Tukey post hoc test, *p* < 0.05.

The absolute plant biomass yield (g) did not significantly vary over the course of the experiment although the general trend of the average yield increased with increasing density (one-way ANOVA: F(7) = 1.57, *p* = 0.174; Figure 2a). SGR (d^−1^) exhibited the opposite trend; rates decreased as density increased (one-way ANOVA: F(7) = 8.357, *p* < 0.001; Figure 2b). The overall removal of TN (mg) from synthetic dairy wastewater increased as plant density increased (one-way ANOVA: F(7) = 2.574, *p* < 0.05; Figure 2c). However, when TN removal was expressed per frond surface area (mg N m^−2^ day^−1^), the rate decreased as density increased (one-way ANOVA: F(7) = 9.287, *p* < 0.001; Figure 2d). A similar pattern was found for TP removal in which the overall removal of TP (mg) from synthetic dairy wastewater increased as plant density increased (one-way ANOVA: F(7) = 5.11, *p* < 0.001; Figure 2e). While the TP removal rate per frond surface area (mg P m^−2^ day^−1^) decreased as density increased (one-way ANOVA: F(7) = 8.158, *p* < 0.001; Figure 2f). There was no difference in protein content (% dry duckweed mass) detected in relation to plant density (one-way ANOVA: F(7) = 0.334, *p* = 0.933; Figure 2g).

### 3.2. Stationary Remediation Experiment 2: Growth and Remediation at Low and High Density

**Figure 3:**
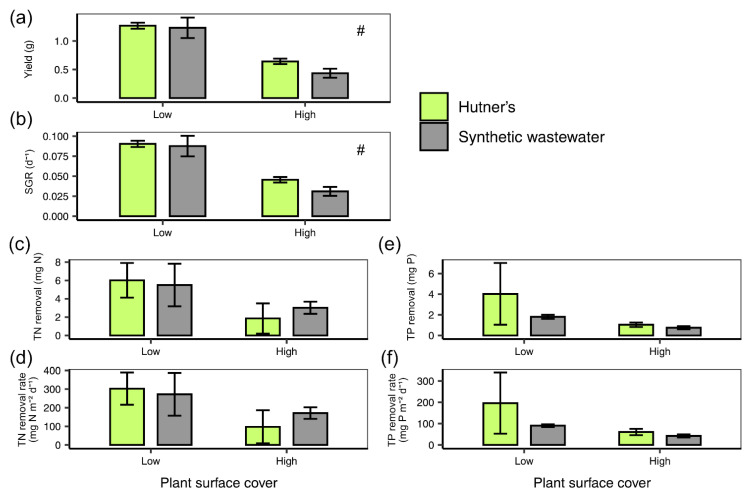
Mean (±SE) (**a**) yield (g), (**b**) SGR (d^−1^), (**c**) TN removal (mg N), (**d**) TN removal rate per frond surface area (mg N m^−2^ d^−1^), (**e**) TP removal (mg P) and (**f**) TP removal rate per frond surface area (mg P m^−2^ d^−1^), for *L. minor* grown on synthetic wastewater or half-strength Hutner’s medium at two plant surface covers (low, 25% and high, 60%) over 7 days. A hash symbol (#) denotes an effect of density for *p* < 0.05, as per the two-way ANOVA.

On both half-strength Hutner’s and synthetic wastewater *L. minor* grown at a lower density (25% plant surface coverage) displayed a higher absolute yield (two-way ANOVA: F(1) = 46.607, *p* < 0.001; Figure 3a) than plants grown at the higher density condition (60% plant surface coverage). The same was found for SGR (two-way ANOVA: F(1) = 45.994, *p* < 0.001; Figure 3b). TN removal (mg) was not significantly affected by density (two-way ANOVA: F(1) = 3.642, *p* = 0.0805; Figure 3c), nor was TN removal rate per frond area (mg N m^−2^ d^−1^) (two-way ANOVA: F(1) = 3.154, *p* = 0.101; Figure 3d), although average values were lower at the higher density. Density condition did not significantly affect TP removal (mg) (two-way ANOVA: F(1) = 2.592, *p* = 0.136; Figure 3e) or TP removal rate per frond area (mg P m^−2^ d^−1^) (two-way ANOVA: F(1) = 2.293, *p* = 0.158; Figure 3f).

### 3.3. Chlorophyll a Fluorescence

**Figure 4:**
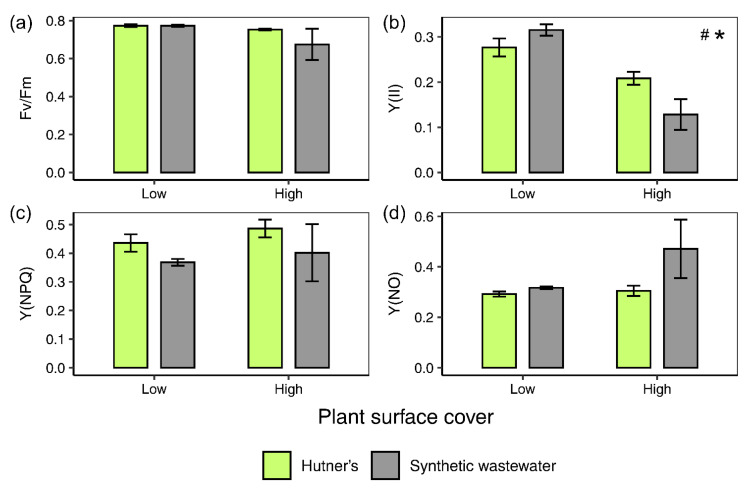
Mean (±SE) (**a**) F_v_/F_m_, (**b**) Y(II), (**c**) Y(NPQ) and (**d**) Y(NO), for *L. minor* grown on either synthetic wastewater or half-strength Hutner’s medium at two plant surface covers (low, 25% and high, 60%) over 7 days. A hash symbol (#) denotes an effect of density for *p* < 0.05, and a star symbol (*) denotes an interactive effect between density and medium for *p* < 0.05, as per the two-way ANOVA.

Chlorophyll *a* fluorescence measurements were taken on the initial day of stationary experiment 2 (day 0, data not shown). They showed that plants grown in both half-strength Hutner’s and synthetic wastewater displayed similar values for a range of chlorophyll fluorescence parameters: F_v_/F_m_, Y(II), Y(NPQ) and Y(NO) (one-way ANOVAs across all treatments: F(1) = 1.006, 0, 0.049, 0.076, *p* = 0.354, 0.986, 0.833, 0.792, respectively). Chlorophyll fluorescence measurements taken on the final day (day 7) of the experiment revealed some differences between treatments. Similar to day 0 values, mean F_v_/F_m_ stayed largely constant between 0.68 and 0.8, and was not significantly affected by density (two-way ANOVA: F(1) = 2.038, *p* = 0.179; Figure 4a) or medium (two-way ANOVA: F(1) = 0.911, *p* = 0.359; Figure 4a). However, measurements taken on day seven showed that higher plant density in both media resulted in a lower Y(II) (two-way ANOVA: F(1) = 34.054, *p* < 0.001; Figure 4b). This effect was strongest in plants grown on synthetic wastewater (post hoc Tukey test low:high density in synthetic wastewater: *p* < 0.001; Figure 4b). Medium alone did not significantly affect Y(II) (two-way ANOVA: F(1) = 0.907, *p* = 0.360; Figure 4b). There was, however, a significant interaction between density and medium (two-way ANOVA interaction ‘density*medium’: F(1) = 7.369, *p* < 0.05; Figure 4b). Y(NPQ) was not significantly affected by density (two-way ANOVA: F(1) = 0.594, *p* = 0.456; Figure 4c) or medium (two-way ANOVA: F(1) = 1.953, *p* = 0.188; Figure 4c). Nor was Y(NO) significantly affected by density (two-way ANOVA: F(1) = 2.016, *p* = 0.181; Figure 4d) or medium (two-way ANOVA: F(1) = 2.631, *p* = 0.131; Figure 4d).

### 3.4. Re-Circulating Remediation System: Growth and Remediation at Variable Plant Densities

**Figure 5:**
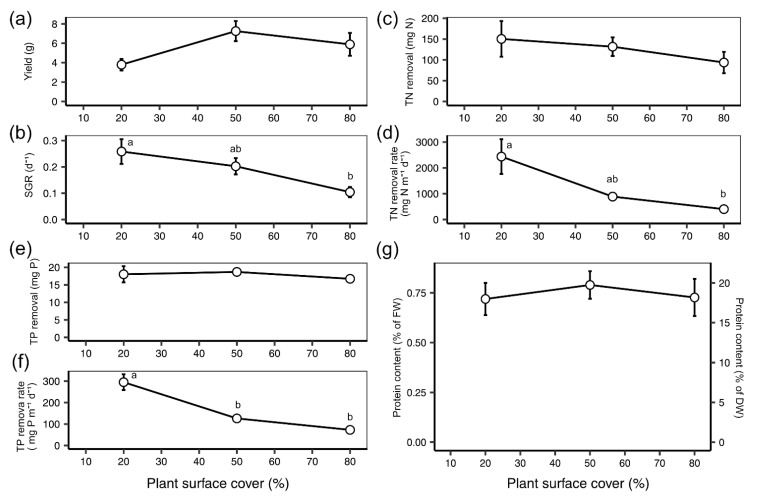
Mean (±SE) (**a**) yield (g), (**b**) SGR (d^−1^), (**c**) TN removal (mg N), (**d**) TN removal rate per frond surface area (mg N m^−2^ d^−1^), (**e**) TP removal (mg P), (**f**) TP removal rate per frond surface area (mg P m^−2^ d^−1^) and (**g**) % protein content based on fresh duckweed biomass, FW, and dry duckweed biomass, DW, for *L. minor* grown on synthetic wastewater in a re-circulating system at three plant surface covers (20, 50 and 80 %) over 5 days. Points that do not share the same letter significantly differ from one another, as per the Tukey post hoc test, *p* < 0.05.

The yield of *L. minor* in the re-circulating experiment was not significantly affected by the plant density (one-way ANOVA: F(2) = 3.238, *p* = 0.087; Figure 5a). However, the average biomass yield was lowest at the 20% plant density and increased to a plateau at 50% (post hoc Tukey: *p* = 0.076; Figure 5a). SGR (d^−1^) steadily decreased with increasing density (one-way ANOVA: F(2) = 5.143, *p* = 0.032; post hoc Tukey 20–80: *p* < 0.05; Figure 5b). Overall, TN removal (mg) from synthetic wastewater was not significantly impacted by density (one-way ANOVA: F(2) = 0.698, *p* = 0.525; Figure 5c). However, TN removal rate (mg N m^−2^ day^−1^) decreased significantly as density increased (one-way ANOVA: F(2) = 5.701, *p* < 0.05; Figure 5d). TN removal rate values dropped from around 2500 mg N m^−2^ day^−1^ at 20% plant surface cover to around 500 mg N m^−2^ day^−1^ at 80% (post hoc Tukey: *p* < 0.05; Figure 5d). Overall, mean TP removal remained at around 17 mg across the three plant densities (one-way ANOVA: F(2) = 0.419, *p* = 0.670; Figure 5e). TP removal rate (mg P m^−2^ day^−1^) decreased with increasing density conditions (one-way ANOVA: F(2) = 29.240, *p* < 0.001; Figure 5f). Mean TP removal rate dropped from 300 mg P m^−2^ day^−1^ at 20% to 75 mg P m^−2^ day^−1^ at 80% (post hoc Tukey: *p* < 0.001; Figure 5f). Protein content (% protein of dry duckweed mass) did not vary between plant densities, with mean values remaining between 17–20% (one-way ANOVA: F(2) = 0.225, *p* = 0.803; Figure 5g).

## 4. Discussion

### 4.1. L. minor Growth on Synthetic Wastewater

RGR values recorded in the stationary experiments (based on the first 3-days of growth; 0.03–0.15 d^−1^) are at the lower end of the range found in the literature for duckweed grown on wastewater (0.04–0.3 d^−1^) [24,51,52]. They are also lower than those found for duckweed grown on an optimised medium (0.153–0.519 d^−1^) [26]. This may, in part, be due to the composition of the medium, as duckweed cultivated on wastewater often results in lower growth rates than obtained on optimised growing medium (compare growth rates in Ziegler et al. [26] with Al-Nozaily et al. [51]). As well as this, the relatively low light intensity of 50 µmol m^−2^ s^−1^ used for the stationary experiments may have also contributed to reduced growth rates [53]. Nevertheless, it has been shown that the light saturation point is around 50 µmol m^−2^ s^−1^ for *L. minor* grown on synthetic dairy wastewater [32], although the light saturation point for duckweed on more optimised media is 400–600 µmol m^−2^ s^−1^ [44,54]. Commonly used experimental light intensities range from 85 to 130 µmol m^−2^ s^−1^ [26,52,55], which are in line with OECD guidelines for duckweed toxicity growth inhibition tests [56]. Although, a higher light intensity of 300 µmol m^−2^ s^−1^ was used in the re-circulating experiment this only led to marginally improved growth rates. In both the stationary and re-circulating experiments, SGR decreased with increasing density, which corresponds with general trends noted within the literature [36,37].

### 4.2. Exploring the Mechanism Underlying Density Dependent Changes in Growth Using Chlorophyll Fluorometry

The negative effect of high density on growth may be a result of greater competition for nutrients [57], a quicker depletion of nutrients [58], or self-shading between colonies [59]. Yet, plant-plant competition has also been related to plant neighbour detection, including plant responses such as shade avoidance, root foraging, and use/induction of chemical defences [60,61]. Therefore, it is possible that *L. minor* senses the closeness of other plants and switches to a more defensive growth strategy, the trade-off of which is a reduction in growth rate. To explore the mechanism underlying the observed decrease in SGR with increasing plant surface density, plants were grown at lower and higher plant surface densities, but with identical biomass per medium volume (stationary experiment 2). This experiment confirmed the impediment of growth rate at higher plant surface densities. However, as plants at each surface density had access to the same volume of medium, the data imply that the growth impediment was due to factors other than nutrient depletion. Indeed, this point is further confirmed by the observation that similar growth impediments occurred at high plant density irrespective of whether nutrient rich Hutner’s medium or more oligotrophic dairy wastewater was used.

Analysis of photosynthetic parameters in stationary experiment 2, measured after seven days of growth, showed that at high density the photosynthetic quantum yield of PSII, Y(II), was significantly depressed. A reduction in Y(II) for *L. minor* grown at high density means that the plants were using light energy less efficiently compared to those at a lower plant surface density [62]; an effect which was stronger for plants grown on synthetic wastewater. Y(II), together with Y(NPQ) and Y(NO) account for the partitioning of absorbed light energy in PSII [63], the sum of which equals 1 [64]. Accordingly, a reduction in Y(II) implies a concurrent increase in Y(NPQ) and/or Y(NO). The data reveal non-significant increases in Y(NPQ) at higher plant densities for both media, indicating minor increases in the amount of light energy dissipated in a regulated manner, i.e., through thermal dissipation [63], at these higher densities. Thus, a key finding is a density-dependent decrease in Y(II) which is not matched by clear significant parallel increases in Y(NO) and Y(NPQ), nor a clear effect on F_v_/F_m_. 

Overall, the data indicate that duckweed density can affect aspects of the plant’s metabolism (e.g., carbon assimilation or nitrogen metabolism), rather than having a direct effect on PSII activity (i.e., F_v_/F_m_). As such, this would indirectly reduce photosynthetic yield (Y(II)) and therefore biomass growth [65]. Previously, Kufel et al. [66] described complex changes in plant morphology in *L. minor* grown at different plant densities. While Zhang et al. [67] described morphological responses of *Spirodela polyrhiza* to population density that included decreased frond and root size, as well as increased frond thickness. It is possible that shading is a driver of these changes and induces a shade avoidance response in plants at higher densities [60,61]. Nevertheless, acclimation to shade typically results in increased Y(II) and decreased Y(NPQ) at low measuring light intensities [68,69]. However, the chlorophyll fluorescence data in this study show decreased Y(II) at higher plant densities where shading might potentially have been an issue. Therefore, neither a lack of nutrient nor light supply, two well-advocated explanations, adequately explain the high-density induced impediment of growth. Rather, the data point to plant neighbour detection between *Lemna*-colonies as the most likely explanation of this [60,61]. At present, touch, volatile organic compounds, chemical exudates and possibly even acoustic signals, have all been associated with neighbour detection [70]. Although it is not known to what extent these apply to duckweed, which tend to produce dense mats in natural habitats [35]. Jang et al. [71] reported that *Lemna japonica* may release interfering chemicals via its root systems, although the effects were interspecific. Similarly, Bich and Kato-Noguchi [72] reported on interspecific allelopathic signals from *Lemna minor*. It is less clear how allelopathic signals manifest between neighbouring duckweed colonies of the same species or clone. In some duckweed species high density, or ‘overcrowding’, has been associated with the production of ethylene, a possible early signal for an increasing lack of space which can inhibit growth [73]. Ethylene has been shown in other plant species to an inhibitor of plant growth [74]. Further studies have shown that the overcrowding-stimulated production of ethylene in duckweed is a Ca^2+^ and phytochrome-dependent process [75,76]. A transient increase in cytoplasmic Ca^2+^ is followed by an increase in ethylene production [75]. Nevertheless, further exploration is required to understand how plant signals, such as ethylene and other potentially unknown signals, influence *L. minor* growth and metabolism in high density conditions. 

### 4.3. L. minor Biomass Yield and Protein Content under Variable Density and System Conditions

Given its high protein content [29,77], and usefulness as a biofuel and a source of phytochemicals [28,78], *Lemna minor* biomass is an important by-product of the wastewater remediation process. As such, the absolute biomass yield is an important parameter, as it relates directly to the amount of plant mass available for further use [27]. Previously, high duckweed densities of 60–80% plant coverage (around 160–280 g m^−2^) have been reported to result in maximum yields, in combination with different harvesting regimes [39,79,80]. Data from stationary experiment 1 and the re-circulating experiment show that at higher plant densities the logarithmic relationship between plant density and yield will plateau. Consequently, the yield increment becomes smaller. However, significant differences between density treatments were not found. Therefore, a clear benefit of higher plant density for biomass yield was not detected. The second stationary experiment shows the opposite trend between density and yield. In this experiment the use of the same plant biomass, but with different container surface areas, led to both a higher growth rate and overall yield at low density. Thus, the use of a shallower/wider container for a volume of wastewater would improve duckweed yield, i.e., surface area space is an important factor in duckweed yield. However, consideration would have to be given to the impact of algae on this result, as less duckweed cover tends to increase the light availability to algae [81].

Duckweed density did not affect protein content in the stationary or re-circulating systems. Nevertheless, the protein content of 30–35% on a dry weight basis found in this study compares favourably with literature sources, where duckweed protein contents up to 45% of dry weight have been reported [44]. Although, more commonly reported values are between 20–35% [28,82]. Furthermore, this compares relatively well with the commonly used high-protein feed, soybean (33–49% [83]). It should be noted that the use of the Bradford assay with BSA as a standard can underestimate plant protein content when compared to other techniques [84,85], which may affect comparisons with literature sources. 

The protein content detected for plants grown in the re-circulating system ranged from 17.5–20% of dry weight, which can be considered a low protein content relative to the published range. This demonstrates that scaling up, and the use of circulatory, non-axenic systems may have unexpected consequences. Starch content was not measured in this study, but, as has been observed in some studies, a lower protein content can result in, or be a result of, higher starch content [27]. If low protein content is a consistent problem for duckweed grown on a large-scale, there are established alternative uses for the biomass that do not depend on the protein content, such as biofuel production [86].

### 4.4. Remediation of TN and TP by L. minor from Synthetic Wastewater

The trend of decreasing growth with increasing density was reflected in the relationship between TN/TP removal rate and density. In both stationary and re-circulating experiments, the lower removal rates of TN and TP per plant surface area (g m^−2^ d^−1^) at higher density conditions show that each duckweed colony is removing less nutrients at higher plant densities than those kept at lower densities. The TN and TP removal rates found in the stationary experiments were on the lower end of the wide range of values recorded in the literature: 124–4400 mg N m^−2^ d^−1^ and 14–590 mg P m^−2^ d^−1^ [57,82,87,88,89,90]. This can be explained by the lower growth rates observed in these experiments [91], which are likely to be in part due to the low light intensity used [32], the specific medium [23], as well as density effects on *L. minor* discussed previously. Both TN and TP removal rates from the non-axenic, re-circulating experiment compare well with values from non-axenic systems in literature sources [57,91]. Higher removal rates found in the re-circulating experiment compared to stationary may be mostly explained by the presence of algae and microorganisms, which can account for up to 50–70% of nutrient removal in non-axenic systems [57,88], but also by improved mixing of the medium.

Another important criterium for analysing the effectiveness of a remediation system is the absolute amount of nutrients removed from the remediated wastewater. In stationary experiment 1, a higher density of plants per surface area more effectively takes up nutrients, although the growth and nutrient removal rates per frond are significantly lower [36,37]. As the removal of nutrients started to plateau at higher densities of 50–80%, this suggests that 50–80% represents an optimal duckweed density for dairy wastewater phytoremediation.

There were additional issues encountered in a non-axenic re-circulating system, in which the absolute removal of TN and TP was relatively flat across the three densities. The exact cause of this is uncertain. However, microorganisms were present in substantial amounts at all plant densities in this non-axenic system. Previously, algae were shown to significantly contribute to nutrient removal [57,81], as well as having negative effects on duckweed such as increased decomposition rates [92]. At both high and low density, a high proportion of the absolute removal of TN and TP may be attributed to microorganisms, breaking the direct link between plant growth and nutrient removal (e.g., poor plant growth, but high nutrient removal). Considering wastewater remediation alone, whether remediation is fuelled primarily by duckweed or algae, nutrient removal is the desired outcome. However, if duckweed biomass is to be generated as a valuable by-product for further use, then strong competition for nutrients with non-utilised microorganisms is undesirable. Ideally, the duckweed should be taking up the majority of the nutrients. One way to achieve this is by maximising the duckweed surface density, thereby decreasing algal growth [93]. Overall, these results show that upscaling from laboratory-based, stationary systems to larger scale, recirculatory systems is complex, and that simple extrapolations are not necessarily correct. Accordingly, management of duckweed incubators will need to be informed by the effects of plant density on biomass yield, TN and TP removal, as well as competition with algal species.

## 5. Conclusions

*Lemna minor* can be successfully grown on synthetic dairy processing wastewater, opening the perspective to both remediate and valorise such waste, in accordance with the principles of the circular economy. *Lemna minor* has been shown to produce the best remediation at higher densities (50–80%), even though growth rates and nutrient uptake rates were slowest at these densities. The decrease in growth at high density was linked to a decrease in photosynthetic yield, rather than competition for light or nutrients, which points towards signals from neighbouring colonies as the potential cause of growth restrictions. However, in non-axenic, scaled-up conditions that better reflect an industrial duckweed-based remediation system, the benefits of high density were not as clear. High algal presence led to suppressed duckweed yield and static nutrient removal. Thus, despite the suitability of *L. minor* for valorisation of dairy processing waste, management of wastewater is subject to both interactions between plant density, yield and nutrient removal, as well as complex upscaling effects.

## Data Availability

The datasets used in the current study are available from the corresponding author on reasonable request.
